# Postpartum Pneumomediastinum and Subcutaneous Emphysema: Two Case Reports

**DOI:** 10.1155/2013/735154

**Published:** 2013-02-25

**Authors:** Shivanthi Kandiah, Harish Iswariah, Stephen Elgey

**Affiliations:** ^1^Department of General Surgery, Redland Hospital, QLD 4163, Australia; ^2^Department of General Surgery, Redland Hospital, and University of Queensland, QLD 4163, Australia; ^3^Consultant Obstetrician and Gynaecologist, Department of Obstetrics and Gynaecology, Redland Hospital, QLD 4163, Australia

## Abstract

Spontaneous pneumomediastinum associated with subcutaneous emphysema is a rare condition also known as Hamman's syndrome. It can also be seen postpartum. We present two cases of subcutaneous emphysema associated with childbirth in nulliparous women, both of which resolved spontaneously.

## 1. Introduction

Hamman noted that in 1940, Faust had gathered 130 reported cases of mediastinal emphysema arising during labour from the medical literature [1]. In the last century there have only been around 200 reported cases of Hamman's syndrome during labour, though the incidence is thought to be between 1 in 100,000 and 1 in 2000 vaginal deliveries [2]. It is predominantly seen in nulliparous women.

Redland Hospital is a 138-bed secondary level hospital in southeast Queensland with approximately 2500 births per annum. There are obstetricians, gynaecologists, and general surgeons on the staff. The two cases reported occurred within three days of each other, in two nulliparous women who were nonsmokers and had no preexisting respiratory disorders. Though the condition is usually self-limiting, it is important that obstetricians and general surgeons are aware of this condition and possible consequences.

### 1.1. Case Report 1

A 25-year-old nulliparous Caucasian woman at 40-week gestation with an uneventful antenatal period had an emergency Caesarean section for failure to progress during the second stage of labour. She had been pushing vigorously for 3 hours 16 minutes during the second stage of labour whilst inhaling Entonox. There were cephalopelvic disproportion and extensive caput of the foetal head. A decision was made to proceed to Caesarean section and not to attempt vaginal instrumentation. She received spinal anaesthetic for the procedure, and a healthy girl weighing 3300 g was delivered. 

On the second postpartum day she noted crackly skin over both sides of her neck and the front of her chest whilst applying body lotion. She denied any dysphagia or dyspnoea. However, on further questioning she did report some chest discomfort whilst on the operating table. She had been tolerating a normal diet. 

On examination she was not in respiratory distress, and oxygen saturation was 99% on room air. Blood pressure was 130/80 mmHg and heart rate 72 bpm. She was afebrile. The lungs were clear on auscultation, and air entry was equal bilaterally. There was crepitus on palpation of the upper anterior chest wall and on both sides of her neck up to the mandible. 

Chest radiography revealed an extensive subcutaneous emphysema in the neck, but there was no obvious pneumomediastinum or pneumothorax. The patient refused to have a CT scan, as she was concerned about the radiation dose. She was discharged home on the fourth postpartum day, as she was asymptomatic apart from the palpable crepitus. Over the course of the next three days she was followed up with home visits by the midwife, and the subcutaneous emphysema had completely resolved. She declined further imaging for followup.

### 1.2. Case Report 2

A 30-year-old nulliparous Caucasian woman had a normal vaginal delivery in hospital at 38-week gestation. She had a spontaneous rupture of membranes and onset of labour 10 hours later. First stage of labour was 4.5 hours and second stage 1.5 hours. Although she did not have a prolonged second stage of labour she had been crying out loudly during the labour, as she had declined an epidural anaesthetic and all other forms of analgesia. She delivered a healthy boy weighing 3500 g. 

Her husband noted that she had a swollen neck on the first postpartum day, and relatives who had been speaking with her over the telephone had commented that her voice was altered. The patient reported that when she first stood up after the delivery she had felt a tight sensation in her neck and upper chest which worsened with deep inspiration. However, she denied any shortness of breath. She did have some difficulty swallowing initially and a sore throat but felt that this had improved and was tolerating a normal diet. 

On examination she was not in respiratory distress, and oxygen saturation was 98% on room air. She did not appear pale or cyanosed. Blood pressure was 110/70 mmHg and heart rate 90 bpm. She was afebrile. There was palpable crepitus over both sides of her neck up to the level of the preauricular region on the right. 

**Figure 1:**
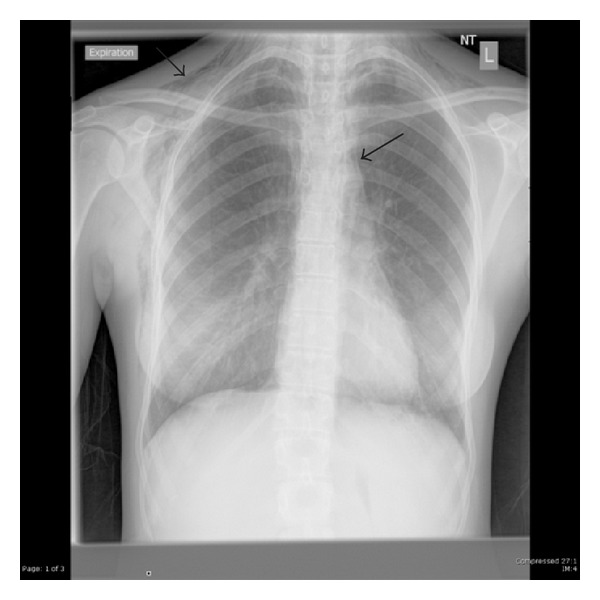
Left pneumomediastinum and subcutaneous emphysema in patient 2.

There was isolated T wave inversion in lead III of the electrocardiogram which is not unusual in pregnancy. Chest X-ray revealed extensive subcutaneous emphysema in the neck and over the right chest wall with a small pneumothorax on the right and minor pneumomediastinum [Figure 1]. Arterial blood gas analysis was normal.

She was transferred to a tertiary centre where she underwent a gastrografin swallow fluoroscopy to exclude oesophageal perforation. The swallow was normal, and she requested discharge from the hospital the next day. However, home reviews by the midwife reported that the subcutaneous emphysema had resolved by day 5 postpartum.

## 2. Discussion

The prolonged Valsalva manoeuvre (straining with the glottis closed) during the second stage of labour as well as the screaming leads to a rupture of marginally situated alveoli into the pulmonary interstitial space, with tracking of air along the bronchovascular connective tissue planes towards the hilum and mediastinum [1].

Subcutaneous emphysema results when air escapes from the mediastinum into the subcutaneous and deep tissues of the neck. When subcutaneous emphysema in the neck and pneumothorax coexist it is likely that both are secondary to mediastinal emphysema, with perforation of the mediastinal pleura resulting in the pneumothorax [1]. 

Malignant pneumomediastinum is the life-threatening condition which is characterized by hypotension and reduced cardiac output due to air under positive pressure remaining trapped in the mediastinal space.

The importance of awareness of this condition, which is usually self-limiting, is to be able to distinguish it from other more life-threatening postpartum conditions such as pulmonary embolism, amniotic fluid embolism, aortic dissection, myocardial infarction, and pneumothorax.

The symptoms include chest pain, dysphagia, odynophagia, dysphonia, dyspnoea, cough, palpitations, anxiety, sore throat, and haemoptysis. The patient may also have a sensation of tearing in the neck which may precede the leaking of air from the mediastinum into the subcutaneous tissues. This may result in crackling sounds. Rarely there may be cyanosis or orthopnoea. There has been a reported case of postpartum hearing loss associated with Hamman's syndrome [3].

Signs of tachycardia, loss of cardiac dullness, decreased heart sounds, and crunching sounds over the praecordium synchronous with the cardiac cycle with systolic accentuation [1, 4] may be elucidated.

The palpation of crepitus in the neck and chest wall raises the suspicion of subcutaneous emphysema. This suggests a more favourable prognosis, as there is a course for air to escape from the mediastinum [5].

Less frequently if pneumopericardium or tension pneumothorax had occurred, there may be cyanosis, venous distension, and signs of cardiorespiratory failure. 

Rarely there may be changes in the ST segment and T wave of the ECG. Arterial blood gas analysis is usually normal, and leucocytosis is common.

A chest radiograph usually reveals the subcutaneous emphysema, and a significant pneumomediastinum or pneumopericardium may be visible. There are no reports of the coexistence of pneumomediastinum with any embolic disorder, and hence confirmation of subcutaneous air and pneumomediastinum on chest radiography obviates the need for further investigations [6].

Computed tomography (CT) is a more accurate radiological tool to reveal pneumomediastinum, and either a CT chest with oral contrast or a gastrografin swallow may be useful to rule out an oesophageal tear (Boerhaave's syndrome). However, this is very uncommon in labour, particularly without a history of vomiting, and as such should only be considered if there is a strong index of clinical suspicion [7]. 

Reassurance and supportive treatment with oxygen, analgesics, and sedatives if the patient is anxious are usually adequate. In the rare event of cardiorespiratory compromise, a mediastinotomy may be necessary to relieve tension pneumomediastinum or symptomatic subcutaneous emphysema. Infraclavicular blowholes in persistent symptomatic subcutaneous emphysema may allow the escape of trapped air [8].

Entonox (nitrous oxide and oxygen) should be avoided if this condition is detected during labour, as it expands the trapped gases. Repeated expulsive efforts should be avoided, and if it is elected to proceed to a Caesarean section, regional anaesthesia is preferable to the positive pressure ventilation involved in a general anaesthetic [9].

The strategy for managing subsequent deliveries is still debated. Some authors have recommended low forceps delivery and avoidance of pushing [10]. However, Seidl and Brotzman have described subsequent uneventful vaginal deliveries [5]. In the absence of strong clinical evidence for preemptive instrumentation, each case should be considered on its own clinical grounds. If spontaneous pneumomediastinum or subcutaneous emphysema is recognized intrapartum, hastening delivery by forceps or Caesarean section may be advisable [11].

It was thought that nulliparity and a prolonged second stage of labour with vigorous expulsive efforts were strongly associated with the condition of intrapartum pneumomediastinum. However, Reeder reviewed 187 cases of Hamman's syndrome in the setting of labour and delivery and concluded that though most women were nulliparous, the second stage of labour was of a normal duration, and the average foetal size was also within normal limits [12].

## 3. Conclusion

The two patients described in this paper were both nulliparous. They had a relatively strenuous and prolonged second stage of labour. Subcutaneous emphysema was noted postpartum.

Pneumomediastinum and subcutaneous emphysema during labour are usually a benign, self-limiting condition. Though uncommon, it needs to be considered as a differential diagnosis, as several conditions with a higher morbidity and mortality share the same clinical presentation.

## References

[1] Hamman L (1945). Mediastinal emphysema. *JAMA*.

[2] Miguil M, Chekairi A (2004). Pneumomediastinum and pneumothorax associated with labour. *International Journal of Obstetric Anesthesia*.

[3] Dilley JWR (2011). Postpartum hearing loss: an unusual presentation of Hamman’s syndrome. *Journal of Obstetrics and Gynaecology*.

[4] Dudley DK, Patten DE (1988). Intrapartum pneumomediastinum associated with subcutaneous emphysema. *CMAJ*.

[5] Seidl JJ, Brotzman GL (1994). Pneumomediastinum and subcutaneous emphysema following vaginal delivery: case report and review of the literature. *Journal of Family Practice*.

[6] Revicky V, Simpson P, Fraser D (2010). Postpartum pneumomediastinum: an uncommon cause for chest pain. *Obstetrics and Gynaecology International*.

[7] Wozniak DR, Blackburn A (2011). Postpartum pneumomediastinum manifested by surgical emphysema. Should we always worry about underlying oesophageal rupture?. *BMJ Case Reports*.

[8] Herlan DB, Landreneau RJ, Ferson PF (1992). Massive spontaneous subcutaneous emphysema; Acute management with infraclavicular ‘blow holes’. *Chest*.

[9] Jayran-Nejad Y (1993). Subcutaneous emphysema in labour. *Anaesthesia*.

[10] Kobak AJ, Abrams RH (1949). Pregnancy complicated by massive subcutaneous emphysema of mediastinal origin (Hamman’s syndrome). *American Journal of Obstetrics and Gynecology*.

[11] Khoo J, Mahanta VR (2012). Spontaneous pneumomediastinum with severe subcutaneous emphysema secondary to prolonged labour during normal vaginal delivery. *Radiology Case Reports*.

[12] Reeder SR (1986). Subcutaneous emphysema, pneumomediastinum, and pneumothorax in labor and delivery. *American Journal of Obstetrics and Gynecology*.

